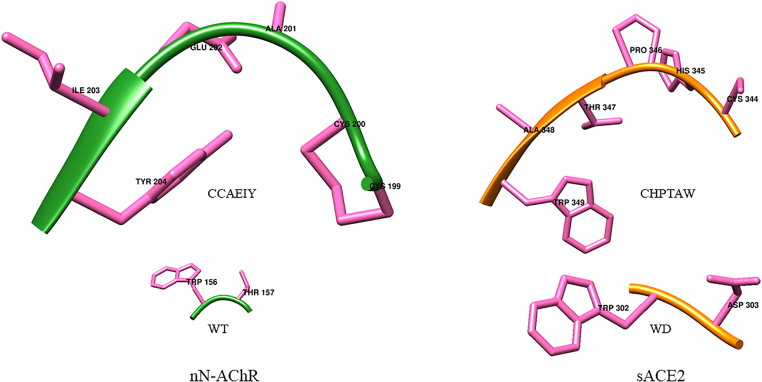# Comparative docking studies to understand the binding affinity of nicotine with soluble ACE2 (sACE2)-SARS-CoV-2 complex over sACE2

**DOI:** 10.1016/j.toxrep.2020.10.002

**Published:** 2020-10-08

**Authors:** Selvaa Kumar C., Senthil Arun Kumar, Haiyan Wei

**Affiliations:** a School of Biotechnology and Bioinformatics, D. Y. Patil Deemed to be University, Sector-15, CBD Belapur, Navi Mumbai, 400614, India; b Department of Endocrinology and Metabolism, Genetics, Henan Children’s Hospital (Children’s Hospital Affiliated to Zhengzhou University), No-33, Longhu Waihuan East Road, Zhengzhou, 450018, China

**Keywords:** Nicotine, Soluble ACE2, Neuronal nicotinic acetylcholine receptor, SARS-CoV-2, sACE2-INS1 complex, Smokers

## Abstract

The study aimed to validate the proficiency of nicotine binding with the soluble angiotensin-converting enzyme II receptor (sACE2) with or without SARS-CoV-2 in the context of its binding affinity. Modelled human sACE2 and the spike (S1) protein of Indian SARS-CoV-2 (INS1) docked with each other. On the other hand, nicotine docked with sACE2 in the presence or absence of SARS-CoV-2. Nicotine established a stable interaction with negatively charged Asp368 of sACE2, which in turn binds with amino acids like Thr362, Lys363, Thr365, Thr371, and Ala372. In the presence of nicotine, INS1 and sACE2 showed a reduced binding affinity score of -12.6 kcal/mol (Vs -15.7 kcal/mol without nicotine), and a lowered interface area of 1933.6 Å^2^ (Vs 2057.3Å^2^ without nicotine). The neuronal nicotinic acetylcholine receptor (nN-AChR) and angiotensin-converting enzyme 2 (ACE2) receptor showed 19.85% sequence identity among themselves. Following these receptors possessed conserved Trp302 and Cys344 amino acids between them for nicotine binding. However, nicotine showed a higher binding affinity score of -6.33 kcal/mol for the sACE2-INS1 complex than the sACE2 alone with -5.24 kcal/mol. A lowered inhibitory constant value of 22.95μM recorded while nicotine interacted with the sACE2-INS1 complex over the sACE2 alone with 151.69 μM. In summary, nicotine showed a profound binding affinity for the sACE2-INS1 complex than the sACE2 alone paving for the clinical trials to validate its therapeutic efficacy as a bitter compound against the SARS-CoV-2 virulence.

## Highlights

• Nicotine established a stable interaction with the conserved amino acids of sACE2 after COVID19 infection.
• Nicotine could not establish proper contact with sACE2 protein in the healthy patients.
• Nicotine reduced the overall binding affinity between sACE2 and RBD domain of SARS-CoV-2 protein.
• Lower dosage of nicotine is required for reducing the binding affinity between sACE2-RBD protein complex.

## Graphical abstract

**Figure:**
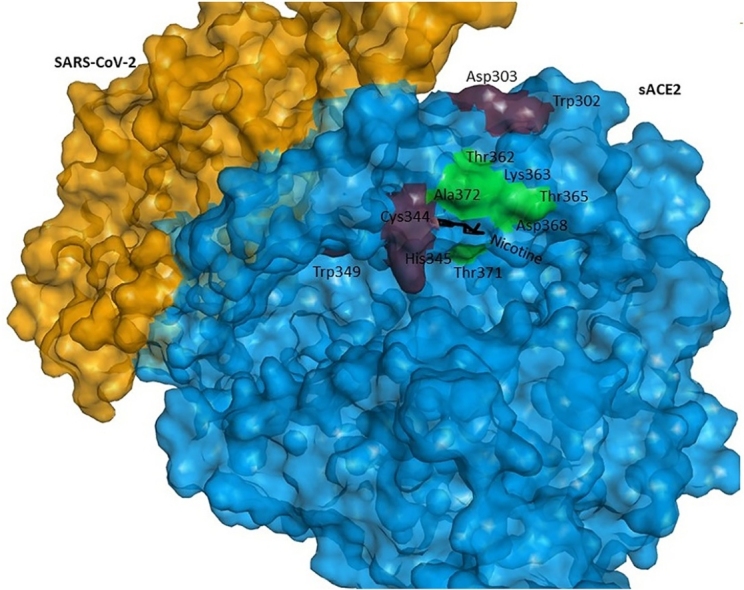

## 1 Introduction

The SARS-CoV-2 pandemic that originated from Wuhan, China has become a dreadful human threat with its unprecedented outbreak [1]. Clinicians and researchers across global countries have abided various clinical strategies to attenuate the SARS-CoV-2 transmission by targeting its virulence characteristics using diverse anti-viral agents, including, the indigenous bio-actives; SARS-CoV-2-protein specific monoclonal antibodies; and convalescent plasma transfusion therapy [[2], [3], [4]]. Angiotensin-converting enzyme II (ACE2) has been the principal target of attenuating the SARS-CoV-2 virulence that acts as the gateway of SARS-CoV-2 (as a receptor agonist) amongst the humans [[5], [6], [7]]. The proliferation of SARS-CoV-2 in the upper respiratory tract has been mediated by its stable interaction with the ACE2 [8]. ACE2 predominantly expressed on the alveolar epithelial cells of lungs; arterial-venous endothelial cells; enterocytes of the small intestine; and arterial smooth muscle cells initiates the conversion of angiotensin-converting enzyme II to I [8,9]. With the increased angiotensin-II levels, SARS-CoV-2 binding with ACE2 perturbs its structural characteristics and biological function preceding the acute lung injury among severely ill patients [9,10]. Acute-lung injury is the outcome of the dysregulated-ACE2 function via SARS-CoV-2 invasion thus triggering the innate-immune response overwhelmingly with the increased pro-inflammatory cytokines production, especially IL-6, IL-8 and IL-1β [11,12]. In compliance with these adverse clinical outcomes associated with the disrupted ACE2 function via SARS-CoV-2 pathogenesis [9,13], the smokers must be the worst victims of this virus attack with the increased mortality. Surprisingly, the smokers showed mild adverse symptoms compared with the non-smokers whereby the mortality rate remains unchanged between these two groups [[14], [15], [16], [17]]. Indeed, this has gathered a wide attention on the medicinal nicotine, a bitter compound exposure among the smokers could intervene the SARS-CoV-2 virulence and therefore its adverse clinical symptoms [18,19]. We hypothesize that nicotine by interrupting the SARS-CoV-2 binding with the ACE2 could attenuate its binding, irrespective of its other likely effects in reversing the cytokine storm regulated by the α4/α7 nicotinic acetylcholine (cholinergic) receptor expressed on the neuronal, muscles, and immune-macrophage cells [18]. Research studies unveiled the interaction between the structural spike 1 (S1) protein of SARS-CoV-2 with the nicotinic acetylcholine receptors (nAChRs) that are likely to intervene with its biological function [20,21]. Amino acid residues of 381–386 of the S1 protein of SARS-CoV-2 actively indulged in establishing a stable interaction with α9 subunit of nAChRs [20,21]. With this interaction, the SARS-CoV-2 could effectively degrade the immune responses of the invaded host. Nicotine by stably interacting with its receptor agonist nAChRs could profoundly overrule the SARS-CoV-2 binding with nAChRs. Indeed, this could replenish the functioning of the nicotinic cholinergic system disrupted by the SARS-CoV-2 invasion [20,22]. Thus, the in-silico study performed to unveil the nicotine’s urge for binding with the soluble ACE2 with or without SARS-CoV-2 in compliance with its interaction with the known human neuronal alpha4-beta2 nicotine-acetylcholine receptor (nN-AChR). As a result, this would unravel the nicotine’s efficacy in hindering the stable-interaction between SARS-CoV-2 and its receptor agonist ACE2. Also, the study outcome will confer a better understanding of the effect of nicotine as a bitter bio-active to tackle the SARS-CoV-2 binding affirmative with ACE2.

## 2 Materials and methods

### 2.1 Protein structure modelling and characterization of soluble ACE2 and spike 1 (S1) protein of SARS-CoV-2 of India origin (INS1)

The human soluble angiotensin-converting enzyme 2 (sACE2) protein sequence was downloaded from the Uniprot database (Accession Number: Q9BYF1) [23]. ACE2 exists in whole (amino acid (aa): 18–805) and in soluble form (sACE2) (aa: 18–708). sACE2 possessed three major domains such as extracellular domain (aa 18–740), helical domain (aa 741–761), and a cytoplasmic domain (aa 792–805). Precisely, the amino acids of ACE2 (aa 30–41; aa 82–84; aa 353–357) would be targeted by the spike 1 (S1) protein of SARS-CoV-2 during the host-invasion via ACE2 binding. We found the reported structural protein template of ACE2-SARS-CoV-2 complex with the enlisted PDB ID: 6VW1 [24] incomplete with numerous missing amino acid residues. The reported ACE2-SARS-CoV-2 complex possessed 614 residues of ACE2 and 527 residues of SARS-CoV-2. For this reason, we performed the homology modelling of each of ACE2 and S1 protein of SARS-CoV-2 study proteins of the ACE2-SARS-CoV-2 complex over utilizing this available crystal ACE2-SARS-CoV-2 complex structure for the study. From the Protein Data Bank [25], the crystal structure of ACE2 with PDB ID: 6m18 [26] with 814 amino acids downloaded with reported missing residues [25,26]. In compliance with the obtained ACE2 template, the three-dimensional (3D) structure of ACE2 developed using SWISS-MODEL [27]. Furthermore, sACE2 region extracted from the modelled ACE2 protein using CHIMERA, which later considered for detailed-structural analysis. Protein sequence (MT012098) [28] of the spike 1 (S1) of SARS-CoV-2 of Indian origin (INS1) was obtained from NCBI and utilized for its 3D-homology modelling using SWISS-MODEL online server. The INS1 protein sequence modelled using PDB ID: 6VSB as a template. This 3D structure showed the sequence identity of 99.17% and a query coverage of 95% [29]. The 3D-modelled protein structures of sACE2 and INS1 were subjected to energy minimization using CHIMERA [30]. Collectively, the structural evaluation of the modelled sACE2 and INS1 performed using PROCHECK-Ramachandran plot server [31]. The in-silico modelled protein structures of the study proteins: ACE2 and S1 protein of SARS-CoV-2 were superimposed with each of its existing crystal structures of ACE2-SARS-CoV-2 complex to elucidate its structural variations (Supplementary Fig. 1a,b).

### 2.2 sACE2 docking with the INS1

Structurally evaluated sACE2 and INS1 protein models considered for docking using HADDOCK server [32]. Based on the Uniprot report, the chosen interface regions of the 3D modelled protein for protein-protein docking lies within the amino acid ranges: 30–41; 82–84; and 353–357. Details regarding “*active site”* residues of INS1 obtained from the literature review [33,34] which form the whole receptor-binding domain (RBD) with the amino acids strecthing from 319−541. Passive amino acids were selected automatically using the checkbox option for sACE2 and INS1. Amino acids proximal to the active site regions chosen as passive residues. Wholly, ten clusters of four poses each generated by the HADDOCK server. Out of ten clusters, the least HADDOCK scored poses were subjected to binding energy evaluation using PDBePISA [35]. Discovery Studio Visualizer standalone software was used to study the docked poses and the interactions [36].

### 2.3 Nicotine docking with the model of INS1 in complex with sACE2

The 3D structure of nicotine downloaded from PDB ID: 1UW6, which later docked with modelled sACE2 protein [37] using AutoDock tools (version 1.5.6) [38]. Based on the previous studies; we found Arg273, His345, Pro346, Glu375, His505, and Tyr515 amino acids as the active-interacting residues of SARS-CoV-2 with sACE2 [34]. Kollman and Gasteiger charges were added to both protein and ligands, respectively. Grid box confined to the active site amino acids. Prior to docking the grid box was generated with a size of 44 Å, 116 Å, and 48 Å for x, y, and z, respectively. Additionally, grid centre values were maintained at -0.861, -6.417, and 3.778, and customized for x, y, and z, respectively. Aside, grid maps developed using Auto Dock 4.0 and Auto Grid 4.0 program. Based on the Lamarckian Genetic Algorithm, ten conformers were generated. Furthermore, for protein-ligand docking, the sACE2-SARS-CoV-2 complex was considered wherein the grid box developed within the sACE2 site with the measured size values of 72 Å, 44 Å, and 82 Å for x, y, and z, respectively. The customized grid centre was at 42.694, -9.11, and 1.389 for x, y, and z, respectively. Concomitantly, the associated grid maps developed using Auto Grid 4.0 and AutoDock 4.0. The region of interaction and the poses visualized using Discovery Studio Visualizer [29].

## 3 Results

### 3.1 Salient structural components of the modelled sACE2 and INS1

ACE2 protein modelled using SWISS-MODEL online server wherein Protein Data Bank ID: 6m18 listed as a potential structural template with 100% amino acid identity and 99 % query coverage. Structurally modelled ACE2 possessed both extracellular and helical domains with the amino acid length of 21–768. Structural analysis by Ramachandra Plot confirms that 91.4% amino acids are in the favoured region; 8.2% in the additional allowed region; 0.4% in the generously allowed region and 0% in the unfavourable region. Thus, sACE2 model with the total size of 21–708 residues extracted from the complete modelled structure.

The 3D-modelled INS1 consists of 27–1146 amino acids classified into S1 and S2 domain. The S1 domain ranges about 27–541, while, the S2 domain ranges about 778−1213. Collectively, the modelled INS1comprised of the N-terminal domain (NTD) (amino acid 27-305); RBD (amino acid 319–436 and amino acid 509–541); receptor binding motif (amino acid 437–508); fusion peptide (amino acid 788–806); heptad repeat region (amino acid 912–984); and the partial heptad repeat region 2 (amino acid 1163–1213). As per the Ramachandran plot report, the core region comprised of 86.1% residues; extended allowed region with 11.9% residues; generously allowed region with 1.7%; and the outlier was 0.3%.

### 3.2 Structural conformations of INS1 interaction with sACE2 in the presence or absence of nicotine

Among the ten clusters developed upon docking of RBD-INS1 with sACE2, cluster with the significantly lowered HADDOCK scores chosen for the next level of screening. Both the structures investigated for their overall interactions. In total, there were eleven hydrogen bonds and three salt bridges observed between INS1 and sACE2. Crucially, all charged amino acids of sACE2 facilitated the interface binding with INS1. A detailed structural examination performed as recorded in our early study [39]. INS1 showed the binding affinity score of -15.7 kcal/mol for sACE2 with the measured interface area of 2057.3 Å^2^. Nonetheless, in the presence of nicotine, the binding-affinity score of INS1 drastically reduced to -12.8 kcal/mol for sACE2 with the decreased interface area of 1933.6 Å^2^.

### 3.3 Effect of nicotine binding on INS1 bound sACE2 protein complex

**Fig. 1:**
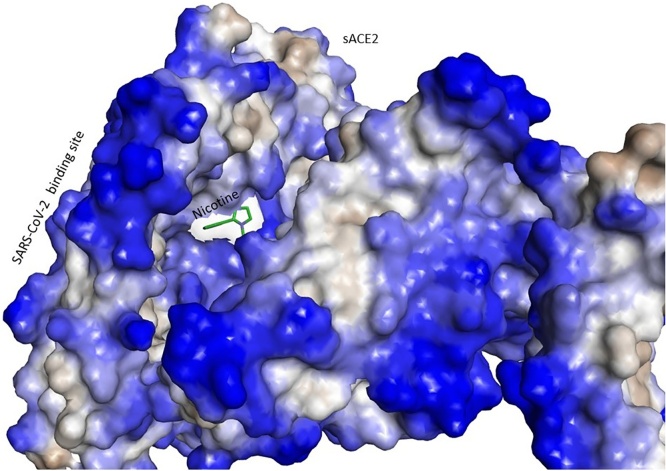
Nicotine docking with the soluble angiotensin-converting enzyme 2 (sACE2) receptor. Nicotine got bound into the active site of sACE2 located near to the SARS-CoV-2 binding site.

**Fig. 2:**
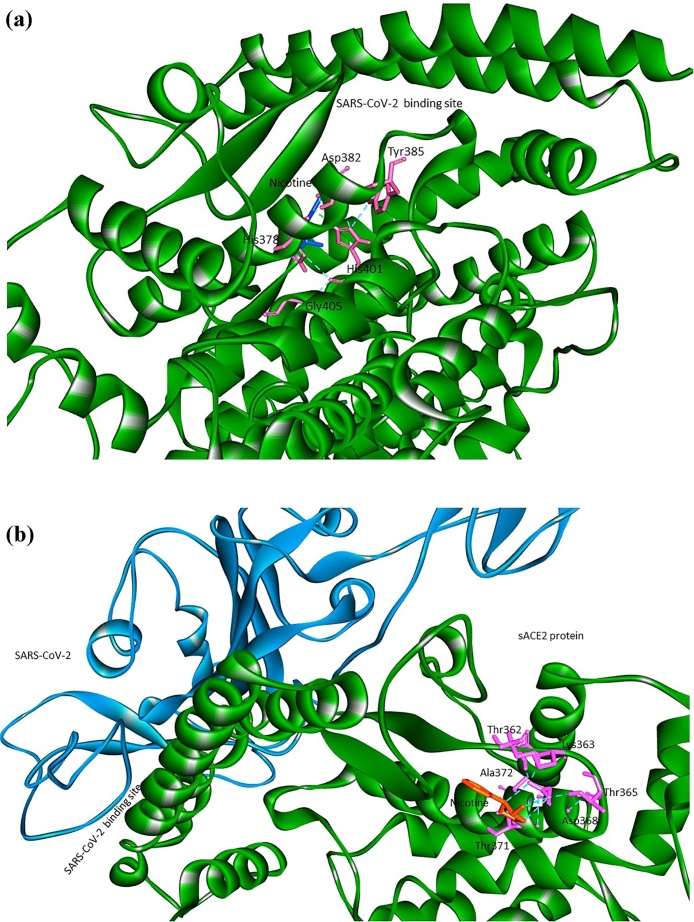
Nicotine docking with the soluble angiotensin-converting enzyme 2 (sACE2) and sACE2-SARS-CoV-2 complex. (a) Nicotine bound intensely into the active site pocket of sACE2 by establishing stable interactions with Asp382, Tyr385, His378, Gly405 through His401 away from the SARS-CoV-2 binding site. (b) Nicotine bound more firmly into the active site pocket of sACE2-SARS-CoV-2 complex (mimicking the diseased condition) by establishing the stable interactions with the Ala372, Thr362, Lys363, Thr365, and Thr371 residues through Asp368 in proximal to the spike (S1) protein binding site of SARS-CoV-2.

Nicotine docking within the conserved-active region of the 3D-modelled sACE2 performed using AutoDock, employs a customized docking module. Nicotine established a stable interaction in the active- site pocket of sACE2 (Fig. 1a). The positively charged His401 of sACE2 sealed its interaction with the nicotine. His401 also developed interactions with the Asp382, Gly405, His378, and Tyr385 residues located in proximity to the distal region of the protein-protein interface (Fig. 2a).

Interaction of nicotine with the INS1 bound sACE2 complex initiated binding with Asp368 residue that strengthens its interactions to the next level of binding with Thr362, Lys363, Thr365, Thr371 and Ala372 residues (Fig. 2b). These supportive five amino acids spotted at the proximal residue site with 353–357 amino acid of sACE2; that engaged in establishing a stable interaction with INS1. Both these sites come opposite to each other (Supplementary Fig. 2). Nicotine exhibited a reduced binding affinity score of -5.24 kcal/mol and a higher inhibition constant of 151.69μM for sACE2 than the INS1 bound sACE2 complex with the binding affinity score of -6.33 kcal/mol and inhibition constant of 22.95μM. From the experimental perspective, the Kd value between ACE2 with SARS−COV-2 is ∼15 nM [29].

Nicotine established a stable interaction within the active site pocket of sACE2. Alternatively, INS1 interacted at the S1 protein binding residue site of sACE2. Collectively, nicotine showed a higher binding affinity with the lowered inhibition constant for the sACE2-INS1 complex over sACE2 alone. This inference unveils the keenness of nicotine binding with the INS1 bound sACE2 complex than sACE2. Moreover, with this profound interaction, it is more likely for nicotine to interrupt the SARS-CoV-2 interaction with ACE2.

### 3.4 Nicotine binding preference for sACE2 with or without INS1 in the context of its interaction with the neuronal nicotinic acetylcholine receptor (nN-AChR) agonist

**Fig. 3:**
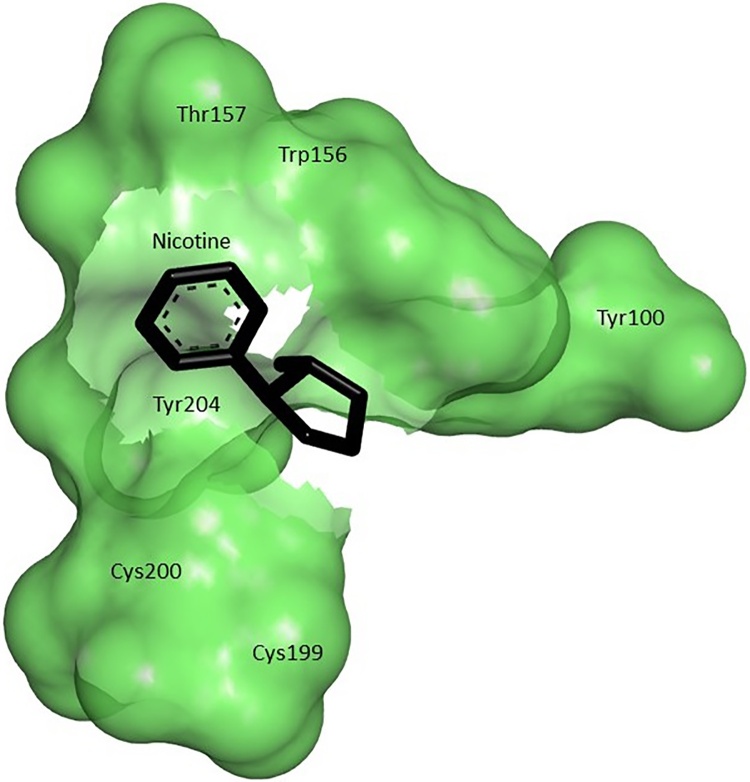
Nicotine binding (in black coloured stick structure) with the human neuronal alpha4-beta2 nicotine-acetylcholine receptor (in green coloured surface structure) with the key amino acid residues engaged in its interaction shown in the surface view.

Crystal structure of the human neuronal alpha4-beta2 nicotinic receptor bound with the nicotine (ligand) procured from the Protein Data Bank (PDB ID: 5KXI) [40]. Based on the PDBsum report, nicotine established a profound interaction with the nN-AChR through amino acids such as Tyr100, Trp156, Cys199, Cys200, and Tyr204 (Fig. 3a) with a binding affinity of 8.3nM from an experimental perspective. These crucial interacting residues were mapped on the modelled human sACE2 by the Clustal Omega-pairwise alignment [41]. As per the alignment report, the overall amino acid identity between sACE2 and nN-AChR was 19.85%. Their global alignment analysis has unravelled the whole 109 identical residues accompanied by the 102 semiconservative residues, and 74 weakly conservative residues (Supplementary Fig. 3a). Two dimensional (2D) structural comparison between the conserved motifs in nN-AChR and sACE2 revealed that amino acids like “CCAEIY” from nN-AChR and “CHPTAW” from sACE2 are a sheet with a short loop region. Conversely, the amino acids: “WT” and “WD” of nN-AChR and sACE2, respectively are short loop region (Supplementary Fig. 3b)

**Fig. 4:**
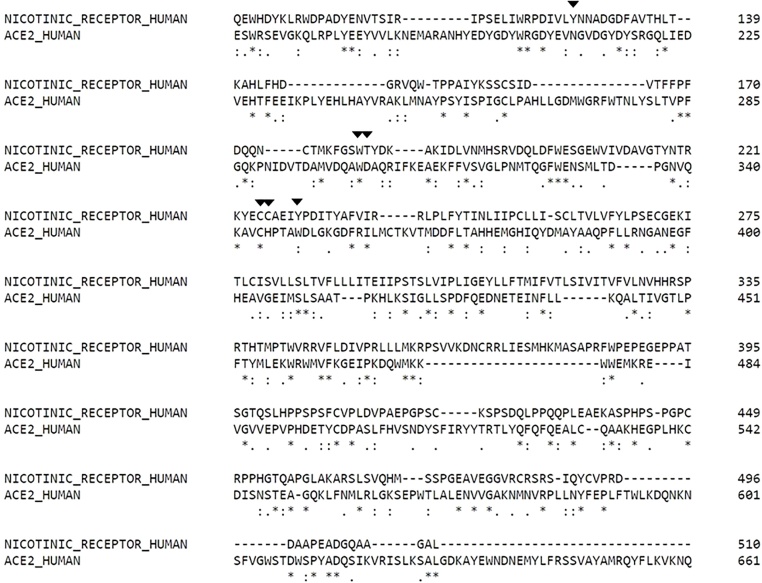
Pairwise sequence alignment profile of the soluble angiotensin-converting enzyme 2 (sACE2) with the neuronal nicotinic acetylcholine receptor (nN-AChR). Paired residues that are exclusively engaged in nicotine binding with the nN-AChR has been marked using the downward arrows on the profile template. The Trp302 and Cys344 of sACE2 residues are highly conserved in compliance to the Trp 156 and Cys 199 residues of nN-AChR. Also, the Trp349 residue of sACE2 and its alternative Tyr204 residue of nN-AChR shared similar characteristics in nature.

**Fig. 5:**
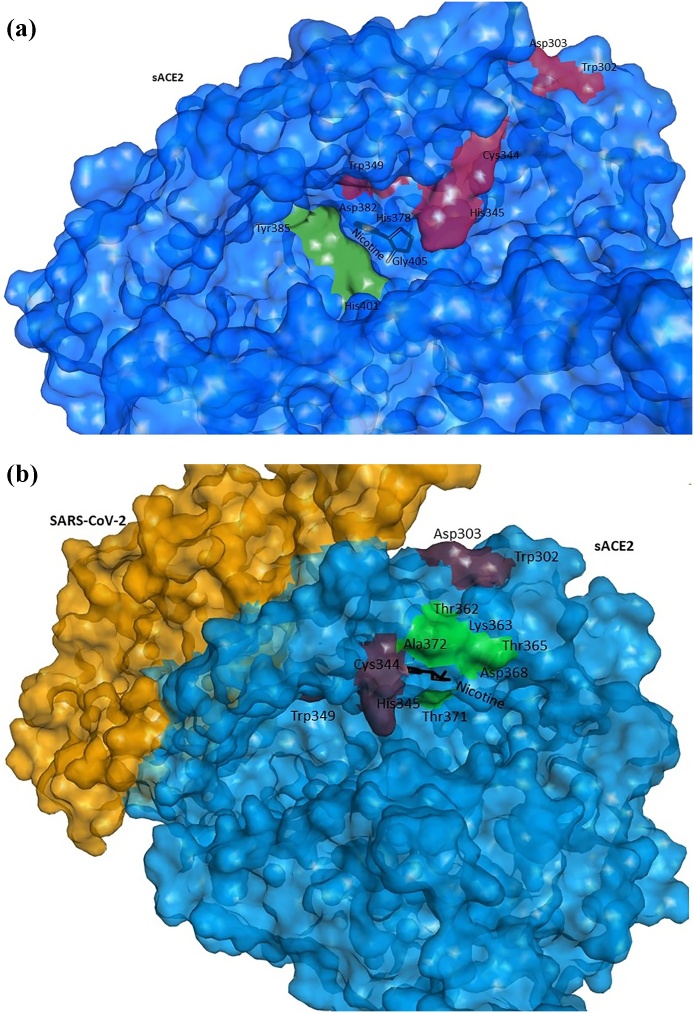
**(a)**Structural binding characteristics of nicotine with the soluble angiotensin-converting enzyme 2 (sACE2) in compliance with its interaction with the neuronal nicotinic acetylcholine receptor (nN-AChR). Conserved residues such as Trp349, Cys344, and His345 of nN-AChR (highlighted in maroon colour) shares proximity with the sACE2 (highlighted in green colour). The conserved residues underlie the stable interaction of nicotine with the sACE2 residues through His401. Asp303 and Trp302 residues of nN-AChR are located far away from the nicotine binding site (highlighted using the maroon colour). **(b):** Structural binding characteristics of nicotine with the soluble angiotensin-converting enzyme 2 (sACE2)-SARS-CoV-2 complex in the context of its interaction with the neuronal nicotinic acetylcholine receptor (nN-AChR). Nicotine bound proximal to the conserved Cys344 residue (marked in maroon colour) of the sACE2-SARS-CoV-2 complex which in-turn facilitates its further interaction with the other flanking residues of sACE2 through Asp368 (marked in green colour).

Surprisingly, the vital nicotine interacting residues of nN-AChR: Trp156 and Cys199 found conserved in sACE2, but with the varied residue numbers of Trp302 and Cys344, respectively. A noticeable conserved residue substitution of Try204 of nN-AChR with Trp349 spotted in sACE2 (Fig. 4). All crucial nicotine interacting and flanking residues of sACE2 such as His401, Asp382, Gly405, His378 and Tyr385 complies well with the nicotine interacting residues: Trp349, Cys344, and His345 of nN-AChR (Fig. 5a). Nevertheless, compared with nN-AChR, the residues: Asn210, Asp303, and Trp302 were spotted distinctly away from the nicotine interacting residues in sACE2. While the residue Asn210 was undetectable in sACE2 (Fig. 4).

Mapping of nicotine binding residues of nN-AChR on INS1-sACE2 complex showed the nicotine-interacting residues flanked by the Asp368, Thr362, Lys363, Thr365, Thr371 and Ala372 residues located proximally to Cys344 (Fig. 5b). Cys344 (as Cys199 in nN-AChR) plays a significant role in affirming the nicotine interaction in both sACE2 and nN-AChR (Fig. 5a and b). The represented study images generated using the Discovery Studio.

## 4 Discussion

Blocking the entry of SARS-CoV-2 via the host based ACE2 receptor has received more attention among the research communities [42]. To gain access into the host system, ACE2 acts as the entry point of SARS-CoV-2 infiltration in all essential organs of the human physiology, in particular the respiratory system [43,44]. A study report has shown the effective tackling of SARS-CoV-2 pathogenesis using Monoclonal Ig-antibodies developed in specific to the human-ACE2 agonist of SARS-CoV-2 [45,46]. In addition to recruiting ACE-2 as the gateway [47], SARS-CoV-2 by establishing a strong interaction with the host-ACE2 destabilize its structural conformation, and interfere its biological role in converting the angiotensin II to angiotensin I [6,48]. The increased angiotensin II levels with the dysregulated ACE2 receptor functions could inflict the acute lung injury among the SARS-CoV-2 patients [49,50]. Smokers being highly vulnerable to SARS-CoV-2 virulence associated with the increased airway-ACE2 expression than the non-smokers [51], incredibly, they showed moderate adverse-clinical symptoms of SARS-CoV-2 than the non-smokers [14,16,18]. This noticeable clinical outcome witnessed among the smokers has compelled us [14,16] to seek for the significant therapeutic action of bitter-bioactive nicotine compound to attenuate the SARS-CoV-2 virulence in the context of its interaction (as an agonist) with the ACE2 receptor [15,52]. The protein-protein docking was performed between nicotine and the 3D-modelled sACE2 protein to understand the crucial nicotine-interacting residues of sACE2. In this study, nicotine’s preference for the sACE2 binding resembles its binding affinity characteristics for the known nN-AChR [[53], [54], [55]] whereby the conserved Trp302 and Cys344 residues flanked by the other crucial His378, Asp382, His401,Gly405, and Tyr385 residues have predominantly determined its interaction with the sACE2 (Figs. 2a and 4). Overall, the interaction of nicotine was wrapped by the crucial positively charged His401 through which the other noticeable bindings with His378, Asp382, Tyr385 and Gly405 residues of sACE2 were established (Fig. 2a). Concomitantly, the study examined the effect of nicotine binding on the SARS-CoV-2 bound sACE2 complex. In comparison with standalone sACE2, nicotine exhibited a higher binding affinity with the lowered inhibitory constant for the sACE2-INS1 complex, that has been facilitated by the conserved Cys344 residue (in compliance to Cys199 of nN-AChR) flanked by the Thr362, Lys363, Thr365, Asp368, Thr371 and Ala372 residues of sACE2-INS1 complex (Fig. 5). Moreover, the positively charged nicotine primarily interacts with the negatively charged Asp368 of the sACE2-INS1 complex through which the nicotine binding got strengthened by interacting with the other Thr362, Lys363, Thr365, Thr371, and Ala372 residues (Fig. 2b). Synergistically, both aspartic acid and threonine residues stabilized the nicotine binding with the sACE2-INS1 complex. The study inference complies with the literature review [37]; that unveiled the nicotine binding affirmative for the aspartic acid, a highly conserved residue spotted at the ligand-gated ion channels superfamily of nicotinic acetylcholine receptors crucial for its structural stability. Additionally, threonine also plays a vital role in strengthening the nicotine binding with the nicotinic acetylcholine receptors. With the nicotine being interacting exclusively within the active protein site (Thr362, Lys363, Thr365, Asp368, Ala372, and Thr371) of sACE2 located right opposite to the INS1 binding residues (Arg357, Asp355, Phe356, Gly354, and Lys353) (Fig. 1b); we hypothesize that nicotine may exhibit an allosteric effect on the INS1 binding with sACE2.

To summarize, nicotine’s better binding preference for the INS1 bound sACE2 complex than the naïve sACE2 alone prone to interlude the binding of sACE2 with SARS-CoV-2. Undeniably, this could be the likely mechanism of nicotine exposure to attenuate the adverse clinical symptoms of SARS-CoV-2 patients who habituated to smoking over the non-smokers. We are intensely cautious about this study, wherein we don’t encourage nicotine exposure via injurious cigarette smoking among the population as it is detrimental to lungs and health. The study outcomes merely emphasize the likely-benefits of nicotine as a bitter compound to counter the adverse clinical symptoms of SARS-CoV-2 pathogenesis by intervening the profound interaction of SARS-CoV-2 with ACE2; that further demands an in-detail clinical validation among the SARS-CoV-2 patients. Medicinal nicotine referred for the treatment of various neurological disturbances [56,57], could be utilized to treat the SARS-CoV-2 patients after being clinically evaluated of its therapeutic effects of dose/usage under the recommended safety-guidelines [15,18,19].

## Author statement

SKC and SAK: Contributed equally to this manuscript. They analysed data, generated figures and helped in writing with the manuscript. HW: Reviewed the manuscript.

## Declaration of Competing Interest

The authors report no declarations of interest.

## Figures and Tables

**Figure:**
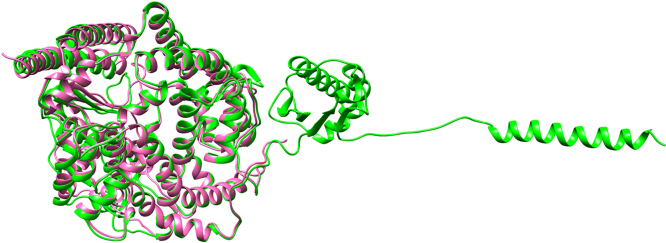


**Figure:**
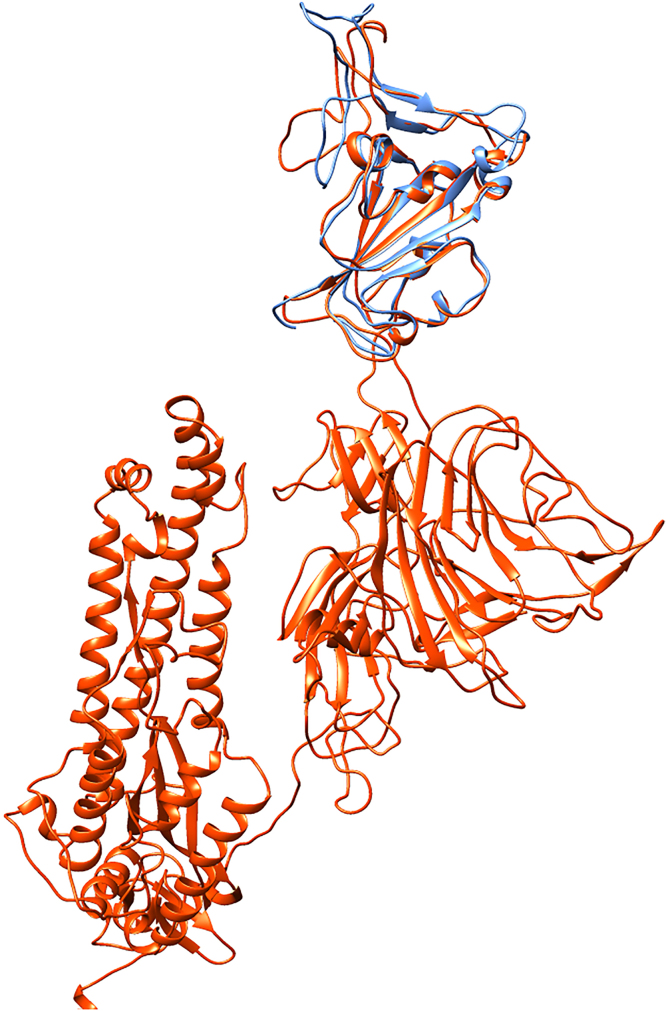


**Figure:**
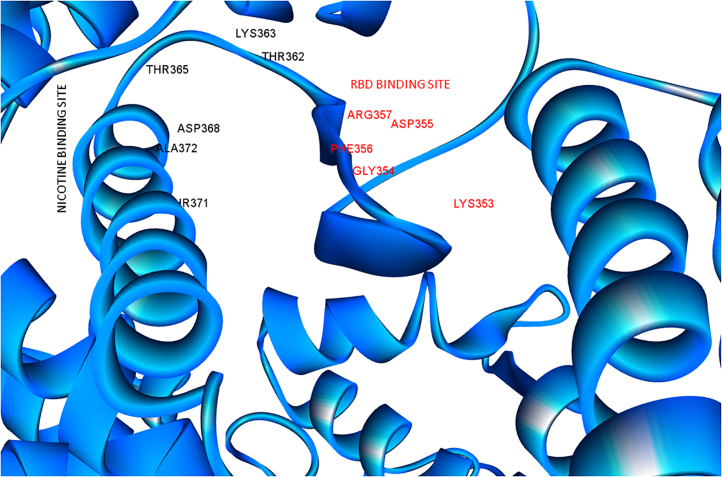


**Figure:**