# Supplementary material for: Comparative docking studies to understand the binding affinity of nicotine with soluble ACE2 (sACE2)-SARS-CoV-2 complex over sACE2
Source: Toxicol Rep. 2020 Oct 8;7:1366–72. doi: 10.1016/j.toxrep.2020.10.002 (PMC7543737; doi:10.1016/j.toxrep.2020.10.002)
Supplement: Supplementary file 1 [file mmc1.docx]

**Supplementary figures**

1. Structural illustration of the superimposed in-silico modelled protein structures of angiotensin-converting enzyme 2 (ACE2) and spike 1 (S1) protein of SARS-CoV-2 with each of its crystal protein structures of the reported ACE2-SARS-CoV-2 complex (PDB ID: 6VW1). (a)The 3D modelled ACE2 protein (in green colour) possessed higher amino acid residues of 768 compared with the reported 614 residues of the ACE2 crystal structure (in pink colour) of ACE2-SARS-CoV-2 complex. (b) While the modelled S1 protein of SARS-CoV-2 (in orange colour) also possessed higher amino acid residues of 1146 compared with the reported 527 amino acid residues of S1 protein crystal structure (in blue colour) of the ACE2-SARS-CoV-2 complex.

2. In soluble angiotensin-converting enzyme 2 (sACE2) protein, the binding site of Receptor Binding Domain (353-357) (labelled in red colour text) is located opposite to the nicotine binding site (labelled in black colour text)

3(a)The pairwise alignment of the human nicotinic acetylcholine receptor (nN-AChR) with human angiotensin-converting enzyme 2 (ACE2) protein. Highly conserved residues shown in Asterix (*) spreading from the N-terminal to the C-terminal regions. The residues with similar physicochemical properties got evenly distributed between these two sequences. The noticeable-similar residues with weaker physicochemical properties marked using a single dot (.) have spotted within the pairwise alignment. Collectively, the alignment confirms many short amino acid segments as conserved residues between these two protein sequences.

3(b) The secondary structural comparison of conserved motifs between the neuronal nicotinic acetylcholine receptor (nN-AChR) and soluble angiotensin-converting enzyme 2 (sACE2) protein showed common sheet and loop regions.
