# Supplementary material for: Comparative docking studies to understand the binding affinity of nicotine with soluble ACE2 (sACE2)-SARS-CoV-2 complex over sACE2
Source: Toxicol Rep. 2020 Oct 8;7:1366–72. doi: 10.1016/j.toxrep.2020.10.002 (PMC7543737; doi:10.1016/j.toxrep.2020.10.002)
Supplement: Supplementary file 2 [file mmc2.pdf]

CLUSTAL O(1.2.4) multiple sequence alignment

```
NICOTINIC_RECEPTOR_HUMAN      MELGGPGAPRLLPPLLLLLG-TGLLRASSHVETR--------AHAEERL-----------   40
ACE2_HUMAN                    ----------MSSSSWLLLSLVAVTAAQSTIEEQAKTFLDKFNHEAEDLFYQSSLASWNY   50
                                        :     ***. ..:  *.* :* :         *  * *

NICOTINIC_RECEPTOR_HUMAN      --------LKKLFSGYNKWSRPVA------------------------------------   56
ACE2_HUMAN                    NTNITEENVQNMNNAGDKWSAFLKEQSTLAQMYPLQEIQNLTVKLQLQALQQNGSSVLSE   110
                                      :::: .. :***  :

NICOTINIC_RECEPTOR_HUMAN      --------------------------NISDVVLVRFGLSIAQLIDVDEKNQMMTTNVWVK   90
ACE2_HUMAN                    DKSKRLNTILNTMSTIYSTGKVCNPDNPQECLLLEPGLNEIMA-----NSLDYNERLWAW   165
                                                        * .: :*:. **.         :.   . .:*.

NICOTINIC_RECEPTOR_HUMAN      QEWHDYKLRWDPADYENVTSIR---------IPSELIWRPDIVLYNNADGDFAVTHLT--   139
ACE2_HUMAN                    ESWRSEVGKQLRPLYEEYVVLKNEMARANHYEDYGDYWRGDYEVNGVDGYDYSRGQLIED   225
                              :.*:.   :     **: . ::               ** *  : .  . *::  :*

NICOTINIC_RECEPTOR_HUMAN      KAHLFHD-------------GRVQW-TPPAIYKSSCSID---------------VTFFPF   170
ACE2_HUMAN                    VEHTFEEIKPLYEHLHAYVRAKLMNAYPSYISPIGCLPAHLLGDMWGRFWTNLYSLTVPF   285
```

```
                                * *.:             .::    *  *   .*                     .**

NICOTINIC_RECEPTOR_HUMAN      DQQN-----CTMKFGSWTYDK----AKIDLVNMHSRVDQLDFWESGEWVIVDAVGTYNTR   221
ACE2_HUMAN                    GQKPNIDVTDAMVDQAWDAQRIFKEAEKFFVSVGLPNMTQGFWENSMLTD-----PGNVQ   340
                              .*:       :*   :*  ::    *:  :*.:       .***..  .        *.:

NICOTINIC_RECEPTOR_HUMAN      KYECCAEIYPDITYAFVIR-----RLPLFYTINLIIPCLLI-SCLTVLVFYLPSECGEKI   275
ACE2_HUMAN                    KAVCHPTAWDLGKGDFRILMCTKVTMDDFLTAHHEMGHIQYDMAYAAQPFLLRNGANEGF   400
                              *  *    :   .  * *       :  * * :  :  :    . :.  * * . ..* :

NICOTINIC_RECEPTOR_HUMAN      TLCISVLLSLTVFLLLITEIIPSTSLVIPLIGEYLLFTMIFVTLSIVITVFVLNVHHRSP   335
ACE2_HUMAN                    HEAVGEIMSLSAAT---PKHLKSIGLLSPDFQEDNETEINFLL------KQALTIVGTLP   451
                                .:. ::**:.      : : * .*: * : *     : *:         .*.:    *

NICOTINIC_RECEPTOR_HUMAN      RTHTMPTWVRRVFLDIVPRLLLMKRPSVVKDNCRRLIESMHKMASAPRFWPEPEGEPPAT   395
ACE2_HUMAN                    FTYMLEKWRWMVFKGEIPKDQWMKK-----------------------WWEMKRE----I   484
                               *: : .*   ** . :*:   **:                       :*   .

NICOTINIC_RECEPTOR_HUMAN      SGTQSLHPPSPSFCVPLDVPAEPGPSC-----KSPSDQLPPQQPLEAEKASPHPS-PGPC   449
ACE2_HUMAN                    VGVVEPVPHDETYCDPASLFHVSNDYSFIRYYTRTLYQFQFQEALC--QAAKHEGPLHKC   542
                               *. .  * . ::* * .:    .  .     .    *:  *: *   :*: * .    *
```

```
NICOTINIC_RECEPTOR_HUMAN      RPPHGTQAPGLAKARSLSVQHM---SSPGEAVEGGVRCRSRS-IQYCVPRD---------   496
ACE2_HUMAN                    DISNSTEA-GQKLFNMLRLGKSEPWTLALENVVGAKNMNVRPLLNYFEPLFTWLKDQNKN   601
                                 :.*:* *    . * : :    :   * * *. . . *  ::*  *

NICOTINIC_RECEPTOR_HUMAN      -------DAAPEADGQAA------GAL---------------------------------   510
ACE2_HUMAN                    SFVGWSTDWSPYADQSIKVRISLKSALGDKAYEWNDNEMYLFRSSVAYAMRQYFLKVKNQ   661
                                     * :* ** .        .**

NICOTINIC_RECEPTOR_HUMAN      ----ASRNTHSAELPPPDQPSPCKCTCKKEPSSVSPSATVKTRSTKAPPPHLPLSPALTR   566
ACE2_HUMAN                    MILFGEEDVRVANLKPRI-SFNFFVTAPKNVSDIIPRTEVE--------------KAIR-   705
                                  ...:.: *:* *         *. *: *.: * : *:               *:

NICOTINIC_RECEPTOR_HUMAN      AVEGVQYIADHLKAEDTDFSVKEDW-KYVAMVIDRIFLWMFIIVCLLGTV--GLFLPPWL   623
ACE2_HUMAN                    --MSRSRINDAFRLNDNSLEFLGIQPTLGPPNQPPVSIWLIVFGVVMGVIVVGIVILI-F   762
                                 . . * * :: :*..:..     .        : :*::::  ::*.:  *:.:   :

NICOTINIC_RECEPTOR_HUMAN      AGMI---------------------------------------   627
ACE2_HUMAN                    TGIRDRKKKNKARSGENPYASIDISKGENNPGFQNTDDVQTSF   805
                              :*:
```
